# Results of Surgical Operations in Malignant Diseases

**Published:** 1851-08

**Authors:** S. D. Gross

**Affiliations:** University of Louisville


RESULTS OF SURGICAL OPERATIONS IN MALIGNANT DISEASES.

*To the Medical Profession of the United States.*

The undersigned having been appointed, at the last meeting of the American Medical Association, Chairman of the Committee on the "Results of Surgical Operations in Malignant Diseases," respectfully solicits contributions to the subject, founded upon personal observation. To place the subject in as tangible a form as possible, he begs leave to direct attention to the following points:

1. The difference between cancerous and cancroid diseases, or those affections which are truly malignant, and those which are only partially so. In the former category are comprised scirrhus, encephaloid, and melanosis; in the latter, certain maladies of the skin and mucous tissues, as lupus, cheloid, eiloid, and cancer of the lip.

2. The precise seat of the disease, as the skin and subcutaneous cellular tissue; the eye, ears, nose, face, lips, tongue, salivary glands, jaws, and gums; the lymphatic ganglions of the neck, axilla, groin, and other regions; the mammary gland, uterus, ovary, vulva and vagina, penis and testis; the anus and rectum; and, finally, the extremities.

3. The age, sex, temperament, residence, and occupation of the patient.

4. The cause of the disease, its progress, and the state of the part and of the system at the time of the operation.

5. Mode of operation; whether by the knife, caustic or ligature.

6. Time of death, or relapse, after operation.

7. Examination of the morbid product; how conducted—whether by the unassisted eye alone, or by means of the microscope, and chemical tests.

The undersigned hopes that the importance of the subject confided to him, as chairman of the committee above referred to, will be sufficiently appreciated by his professional brethren, to induce them to aid him in carrying out the wishes of the American Medical Association. The subject is one of absorbing interest, and cannot fail, if properly treated, to elicit matter of the greatest benefit. It is very necessary that all communications upon the subject, should be sent to the chairman of the committee by the 1st of January, 1852.

Medical journals, and newspapers friendly to the interests of medical science, will confer a favor upon the undersigned, by inserting the above notice.

S. D. GROSS, M. D.

University of Louisville, June 29, 1851.

